# Projected Trends in Metabolic Dysfunction–Associated Steatotic Liver Disease Mortality Through 2040

**DOI:** 10.1001/jamanetworkopen.2025.16367

**Published:** 2025-06-17

**Authors:** Xinrong Zhang, Sovann Linden, Charles R. Levesley, Xinyuan He, Zhanpeng Yang, Scott D. Barnet, Ramsey Cheung, Fanpu Ji, Mindie H. Nguyen

**Affiliations:** 1 Division of Gastroenterology and Hepatology, School of Medicine, Stanford University Medical Center, Palo Alto, California; 2 Department of Management Science and Engineering, School of Engineering, Stanford University, Palo Alto, California; 3 Department of Biology, Stanford University, Palo Alto, California; 4 Department of Infectious Diseases, The Second Affiliated Hospital of Xi’an Jiaotong University, Xi’an, China; 5 School of Mathematics and Statistics, Xi’an Jiaotong University, Xi’an, China; 6 Veterans Affairs Palo Alto Health Care System, Palo Alto, California; 7 Key Laboratory of Environment and Genes Related to Diseases, Xi’an, Jiaotong University, Ministry of Education of China, Xi’an, China; 8 Department of Epidemiology and Population Health, Stanford University Medical Center, Palo Alto, California; 9 Stanford Cancer Institute, Stanford University Medical Center, Palo Alto, California

## Abstract

**Importance:**

Population-based data for metabolic dysfunction–associated steatotic liver disease (MASLD)–related mortality trends and forecasts in the United States are limited.

**Objective:**

To examine MASLD-related mortality trends in the United States from 2006 to 2023 and forecast mortality rates up to 2040 overall and in subgroups by age, sex, race and ethnicity, and urbanization.

**Design, Setting, and Participants:**

This cross-sectional study used data from the National Vital Statistics System dataset. Data on deaths attributed to MASLD were obtained for adults aged 25 years and older from January 1, 2006, to December 31, 2023.

**Main Outcomes and Measures:**

Trends were evaluated by average annual percentage change (AAPC) in age-standardized mortality rates (ASMRs) per 100 000 persons, and mortality rates were forecasted to 2040 using projection models.

**Results:**

A total of 27 961 decedents aged 25 years and older with MASLD (15 251 [54.5%] aged ≥65 years; 15 450 [55.3%] female; 3373 [12.1%] Hispanic, 1480 [5.3%] non-Hispanic Black, and 21 936 [78.5%] non-Hispanic White) were documented from 2006 to 2023. ASMRs rose from 0.25 to 1.27 per 100 000 persons, with AAPCs increasing from 9.27% in 2006 to 2018 to 22.66% in 2018 to 2021, then decreasing to −1.23% from 2021 to 2023, leading to projected ASMRs of 2.24 per 100 000 persons in 2040. There were significant differences in the increases of ASMRs by age, with those aged 65 years or older having the steepest rise (AAPC, 15.34%; 95% CI, 14.40%-16.32%; *P* < .001; 45-64 years: 8.76%; 95% CI, 7.29%-10.22%; *P* < .001; 25-44 years: 2.65%; 95% CI, 0.49%-4.86%; *P* = .02) and a projected increase from 3.69 per 100 000 persons in 2024 to 7.12 per 100 000 persons in 2040. However, there was no significant difference in ASMRs by sex (AAPC among women: 11.24%; 95% CI, 10.09%-12.40%; *P* < .001; AAPC among men: 11.04%; 95% CI, 9.56%-12.63%; *P* < .001). ASMRs rose for all major racial ethnic groups, with the highest ASMR increase observed for non-Hispanic White individuals (AAPC, 11.12%; 95% CI, 9.48%-12.83%; *P* < .001), followed by Hispanic (AAPC, 10.67%; 95% CI, 9.11%-12.26%; *P* < .001), non-Hispanic Black (AAPC, 9.20%; 95% CI, 7.32%-11.11%; *P* < .001), and non-Hispanic Asian (AAPC, 7.97%; 95% CI, 4.66%-11.75%; *P* < .001) individuals, while the projected values for these 4 groups showed similar increasing trends to 2040. There were also significant differences in ASMRs by metropolitan categories overall, with the highest rise in nonmetropolitan areas (AAPC, 13.50%; 95% CI, 10.70%-16.32%; *P* < .001).

**Conclusions and Relevance:**

In this cross-sectional study, MASLD-related mortality increased rapidly between 2006 and 2023 and was projected to rise over the next 20 years, with the largest disparities among those aged 65 years and older, among non-Hispanic White and Hispanic individuals, and among nonmetropolitan populations.

## Key Points

**Question:**

How did metabolic dysfunction–associated steatotic liver disease (MASLD)–related mortality rates change from 2006 to 2023, and what can be forecasted though 2040 in the United States?

**Findings:**

In this cross-sectional study that included 27 961 adult decedents, the MASLD-related mortality rate increased rapidly from 2006 to 2023 and was projected to continue to rise through 2040. The largest disparities were observed among those aged 65 years and older, Hispanic and non-Hispanic White populations, and nonmetropolitan populations.

**Meaning:**

These findings could inform medical practice and public health to address increased MASLD-related mortality trends and timely identification of high-risk populations for interventions to reduce MASLD-related mortality in the United States.

## Teaser

This cross-sectional study examines the trends in metabolic dysfunction–associated steatotic liver disease (MASLD)–related mortality in the United States from 2006 to 2023 and projects mortality rates by age, sex, race and ethnicity, and urbanization through 2040.

## Introduction

Metabolic dysfunction–associated steatotic liver disease (MASLD) affects approximately 30% of the global population and is the leading cause of chronic liver diseases in the world.^1^ Around 15% of patients with MASLD develop metabolic dysfunction–associated steatohepatitis, which can lead to cirrhosis, hepatocellular carcinoma (HCC), and eventually mortality.^2^

An increasing trend of MASLD-related mortality in the United States from 1999 to 2022 has been reported.^3^ The potential biological mechanisms of increased MASLD mortality rates include increased insulin resistance, chronic inflammation, oxidative stress, and gut dysbiosis, triggering fibrosis, cirrhosis, and HCC development and eventually mortality.^4^ Additionally, the lack of awareness and delayed diagnosis of MASLD, poor linkage to care and limited pharmacologic treatments targeting MASLD may also lead to increased mortality rates.^5^ However, forecasts for MASLD mortality based on population-based data in the United States are limited.

As the population ages, the risk of developing advanced stages of MASLD, such as cirrhosis and HCC, as well as liver-related death increases.^6,7^ However, more evidence is needed to determine the age threshold where MASLD-related mortality would rise. Prior epidemiologic studies have shown significant differences in the prevalence and severity of MASLD by race and ethnicity, which may be related to differences in lifestyle, diet, metabolic and genetic profiles, and social factors (eg, social drivers or determinants of health) among others.^8,9,10^ The impact of sex on the natural history of MASLD is another important consideration. A recent meta-analysis showed that there were no sex-related differences in all-cause and liver-related mortality of MASLD,^11^ while other studies indicated that sex difference existed in mortality rates of adults with MASLD.^12,13^ Urbanization can also impact access to health care, lifestyle, diet, and environmental factors that may contribute to MASLD development.^14,15^ However, the impact of urbanization on MASLD-related mortality for public health planning remains unclear. Therefore, we aimed to evaluate temporal changes in MASLD-related mortality in the United States from 2006 to 2023 and forecast mortality rates through 2040 in the overall US population as well as subgroups of age, sex, race and ethnicity, and urbanization.

## Methods

### Study Design and Population

Data were obtained from the National Vital Statistics System (NVSS) dataset through the Center for Disease Control and Prevention Wide-Ranging Online Data for Epidemiologic Research (CDC WONDER) website. This database collected annual death data of more than 99% of decedents in 50 states and the District of Columbia of the United States. We have used final multiple causes of death data from January 1, 2006, to December 31, 2020, and provisional multiple causes of death data from January 1, 2021, to December 31, 2023. Demographic data, including age, sex, race and ethnicity, and urbanization, were obtained. Institutional review board approval and informed consent form were waived in accordance with the Common Rule, because all data from NVSS are publicly available and completely deidentified. The study followed the Strengthening the Reporting of Observational Studies in Epidemiology (STROBE) guideline.

### Inclusion and Exclusion Criteria

We included data on deaths associated with MASLD among adults aged 25 years and older in the United States from January 1, 2006, to December 31, 2023. We excluded alcohol-associated liver disease, which can also present as hepatic steatosis. We defined MASLD as the contributing cause of death by those who had both steatotic liver disease (K75.8, K76.0) and at least 1 cardiometabolic risk factors including diabetes (E10-E14), hypertension (I10), dyslipidemia (E78), and obesity (E66) recorded using the *International Statistical Classification of Diseases and Related Health Problems, Tenth Revision (ICD-10) *diagnosis code, according to the 2023 MASLD guideline of American Association for the Study of Liver Diseases.^16^

### Statistical Analysis

Demographic characteristics of decedents with MASLD were presented as frequencies with percentages. Age-standardized mortality rates (ASMRs) were developed using the age structure (25 to ≥85 years) from the 2000 US Census Standard Population and the direct standardization method (eMethods in Supplement 1).^17^ We conducted joinpoint regression analysis to determine the annual percentage change (APC) with 95% CIs of each segment for mortality trends from 2006 to 2023 (eMethods in Supplement 1). However, analyses involving urbanization were only conducted until 2020 because the dataset did not provide the urbanization data after 2020.

We forecasted mortality rates to 2040 based on the mortality rates from 2006 to 2023 using 2 time-series forecasting models (Prophet and constructed linear regression model) (eMethods in Supplement 1). The selection of 2 models was based on the distribution of the data and model fitness, which assessed through the root mean squared error (eMethods and eTable 1 in Supplement 1). We performed subgroup analyses by using the following groups: age (25-44, 45-64, and ≥65 years), sex (female and male), race and ethnicity (self-reported as Hispanic, non-Hispanic Asian, non-Hispanic Black, and non-Hispanic White groups; participants who belonged to other racial and ethnic groups were excluded), and urbanization (large and fringe metropolitan [>1 million residents], medium and small metropolitan [250 000-999 999 residents and 50 000-249 999 residents, respectively], and nonmetropolitan areas [<50 000 residents]). These subgroups were assessed because disparities have been observed in MASLD-related mortality among different age, sex, racial and ethnic, and urbanization groups. All analyses were performed using the National Cancer Institute’s Joinpoint Trend Analysis software version 4.9.0.0 and R version 4.4.2 (R Foundation for Statistical Computing). A 2-sided *P* value with the threshold of significance at .05 was used.

## Results

### Study Population and Characteristics

A total of 27 961 decedents aged 25 years and older with MASLD as a contributing cause of death were documented from 2006 to 2023 (Table 1). Those aged 45-64 years and 65 years or older accounted for nearly 90% of the population (45-64 years, 9616 [34.4%]; ≥65 years 15 251 [54.5%]) and female decedents (15 450 [55.3%]) outnumbered males (12 511 [44.7%]). The proportion of deaths in the elderly group increased, but those of middle- and younger aged groups decreased between 2006 and 2023. There were 3373 (12.1%) Hispanic, 557 (2.0%) non-Hispanic Asian, 1480 (5.3%) non-Hispanic Black, and 21 936 (78.5%) non-Hispanic White decedents. Only the proportion of deaths in the non-Hispanic White group increased, and those of other racial and ethnic groups decreased after 2020. By metropolitan areas, 11 106 deaths (39.7%) occurred in large central metropolitan areas compared with 9875 (35.3%) and 5536 (19.8%) in medium and small or nonmetropolitan areas, respectively. The proportion of deaths related to liver transplant increased from 4.5% in 2006 to 5.2% in 2010 but decreased to 1.4% in 2023 (eTable 2 in Supplement 1).

**Table 1.** Characteristics of Decedents With Metabolic Dysfunction–Associated Steatotic Liver Disease as a Contributing Cause of Death in the United States, 2006-2023

Characteristics	Deaths, No. (%)							
	2006-2023 (N = 27 961)	2006 (n = 491)	2010 (n = 657)	2015 (n = 1269)	2020 (n = 2885)	2021 (n = 3319)	2022 (n = 3314)	2023 (n = 3464)
Age, y								
25-44	3094 (11.1)	142 (28.9)	108 (16.4)	175 (13.8)	252 (8.7)	327 (9.9)	243 (7.3)	221 (6.4)
45-64	9616 (34.4)	213 (43.4)	286 (43.5)	510 (40.2)	950 (32.9)	1077 (32.4)	967 (29.2)	926 (26.7)
≥65	15 251 (54.5)	136 (27.7)	263 (40.0)	584 (46.0)	1683 (58.3)	1915 (57.7)	2104 (63.5)	2317 (66.9)
Sex								
Female	15 450 (55.3)	240 (48.9)	377 (57.4)	687 (54.1)	1621 (56.2)	1833 (55.2)	1892 (57.1)	1904 (55.0)
Male	12 511 (44.7)	251 (51.1)	280 (42.6)	582 (45.9)	1264 (43.8)	1486 (44.8)	1422 (42.9)	1560 (45.0)
Race and ethnicity								
Hispanic	3373 (12.1)	53 (10.8)	82 (12.5)	143 (11.3)	389 (13.5)	424 (12.8)	402 (12.1)	403 (11.6)
Non-Hispanic Asian	557 (2.0)	14 (2.9)	12 (1.8)	24 (1.9)	73 (2.5)	62 (1.9)	52 (1.6)	57 (1.6)
Non-Hispanic Black	1480 (5.3)	42 (8.6)	32 (4.9)	54 (4.3)	165 (5.7)	171 (5.2)	142 (4.3)	169 (4.9)
Non-Hispanic White	21 936 (78.5)	375 (76.4)	524 (79.8)	1010 (79.6)	2191 (75.9)	2569 (77.4)	2635 (79.5)	2743 (79.2)
Urbanization^a^								
Large and fringe metropolitan	11 106 (39.7)	231 (47.0)	288 (43.8)	536 (42.2)	1250 (43.3)	1171 (35.3)	1102 (33.3)	1130 (32.6)
Medium and small metropolitan	9875 (35.3)	180 (36.7)	251 (38.2)	461 (36.3)	988 (34.2)	1106 (33.3)	1147 (34.6)	1227 (35.4)
Nonmetropolitan	5536 (19.8)	80 (16.3)	118 (18.0)	272 (21.4)	647 (22.4)	577 (17.4)	587 (17.7)	606 (17.5)

^a^ Metropolitan categories defined as follows: large and fringe central metropolitan areas (>1 million residents), medium and small metropolitan areas (250 000-999 999 residents and 50 000-249 999 residents), and nonmetropolitan areas (<50 000 residents).

### Overall and Forecast Analysis of All-Cause MASLD-Related Mortality

**Figure 1.**
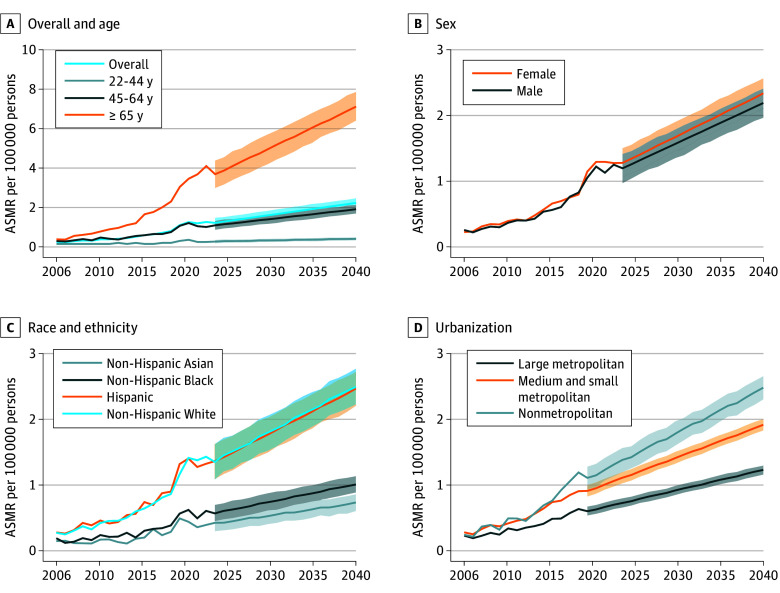
Age-Standardized Mortality Rates (ASMRs) and Projected Values for Metabolic Dysfunction–Associated Steatotic Liver Disease in the United States, 2006-2040 Projections were conducted in the Prophet model.

All-cause ASMR for MASLD increased from 0.25 per 100 000 persons in 2006 to 1.27 per 100 000 persons in 2023. The average APC (AAPC) between 2006 and 2023 was observed as 10.21% (95% CI, 8.65% to 12.13%; *P* < .001) (Table 2; eFigure 1 in Supplement 1). However, on trend segment analysis, APCs accelerated from 9.27% (95% CI, 5.29% to 17.64%; *P* = .02) for 2006 to 2018 to 22.66% (95% CI, 3.97% to 27.92%; *P* = .003) for 2018 to 2021, but the APC decreased at −1.23% (95% CI −13.69% to 15.21%; *P* = .91) from 2021 to 2023. The projected ASMR values of 1.22 per 100 000 persons increased to 2.24 per 100 000 persons from 2024 to 2040 (Figure 1A).

**Table 2.** APC and ASMR Among Decedents With Metabolic Dysfunction–Associated Steatotic Liver Disease as a Contributing Cause of Death, 2006-2023

Characteristic	ASMR, per 100 000 persons							Average APC (95% CI), 2006-2023	*P* value	Trend segment		*P* value
	2006	2010	2015	2020	2021	2022	2023			Year	APC (95% CI), %	
Overall	0.25	0.32	0.52	1.11	1.26	1.20	1.27	10.21 (8.65 to 12.13)	<.001	2006-2018	9.27 (5.29 to 17.64)	.02
										2018-2021	22.66 (3.97 to 27.92)	.003
										2021-2023	−1.23 (−13.69 to 15.21)	.91
Age, y												
25-44	0.15	0.15	0.21	0.31	0.36	0.25	0.25	2.65 (0.49 to 4.86)	.02	2006-2017	0.95 (−4.90 to 5.99)	.59
										2017-2021	18.84 (−2.91 to 33.36)	.07
										2021-2023	−16.03 (−31.39 to 8.46)	.12
45-64	0.30	0.34	0.56	1.08	1.21	1.05	1.01	8.76 (7.29 to 10.22)	<.001	2006-2023	8.76 (7.29 to 10.22)	<.001
≥65	0.39	0.68	1.20	3.05	3.46	3.68	4.10	15.34 (14.40 to 16.32)	<.001	2006-2023	15.34 (14.40 to 16.32)	<.001
Sex												
Female	0.22	0.34	0.57	1.15	1.29	1.29	1.28	11.24 (10.09 to 12.40)	<.001	2006-2023	11.24 (10.09 to 12.40)	<.001
Male	0.26	0.30	0.54	1.05	1.22	1.13	1.25	11.04 (9.56 to 12.63)	<.001	2006-2014	8.64 (−2.26 to 18.91)	.09
										2014-2023	13.22 (0.93 to 25.54)	.04
Race and ethnicity												
Hispanic	0.28	0.39	0.56	1.32	1.40	1.28	1.32	10.67 (9.11 to 12.26)	<.001	2006-2023	10.67 (9.11 to 12.26)	<.001
Non-Hispanic Asian	0.15	0.11	0.18	0.49	0.44	0.36	0.39	7.97 (4.66 to 11.75)	<.001	2006-2014	1.46 (−26.38 to 9.31)	.88
										2014-2023	14.12 (6.18 to 47.34)	.04
Non-Hispanic Black	0.19	0.16	0.20	0.57	0.62	0.50	0.61	9.20 (7.32 to 11.11)	<.001	2006-2023	9.20 (7.32 to 11.11)	<.001
Non-Hispanic White	0.28	0.33	0.60	1.17	1.41	1.38	1.43	11.12 (9.48 to 12.83)	<.001	2006-2014	8.85 (−2.15 to 22.85)	.10
										2014-2023	13.17 (−1.21 to 25.98)	.06
Urbanization^a^												
Large and fringe metropolitan	0.23	0.25	0.41	0.89	NA	NA	NA	10.96 (8.42 to 12.42)	<.001	2006-2018	8.60 (−0.36 to 23.24)	.06
										2018-2020	26.21 (8.07 to 38.52)	.003
Medium and small metropolitan	0.28	0.37	0.64	1.21	NA	NA	NA	10.67 (9.51 to 11.84)	<.001	2006-2020	10.67 (9.51 to 11.84)	<.001
Nonmetropolitan	0.24	0.32	0.69	1.60	NA	NA	NA	13.50 (10.70 to 16.32)	<.001	2006-2020	13.50 (10.70 to 16.32)	<.001

Abbreviations: APC, annual percentage change; ASMR, age-standardized mortality rate; NA, not applicable.

^a^ ASMR did not have data from 2021 to 2023 in urbanization categories. Metropolitan categories defined as follows: large and fringe central metropolitan areas (>1 million residents), medium and small metropolitan areas (250 000-999 999 residents and 50 000-249 999 residents), and nonmetropolitan areas (<50 000 residents).

There were significant differences in the increase of ASMRs by age, with those aged 65 years or older having the steepest rise (AAPC, 15.34%; 95% CI, 14.40%-16.32%; *P* < .001; 45-64 years: 8.76%; 95% CI, 7.29%-10.22%; *P* < .001; 25-44 years: 2.65%; 95% CI, 0.49%-4.86%; *P* = .02) and a projected increase from 3.69 per 100 000 persons in 2024 to 7.12 per 100 000 persons in 2040 (Table 2 and Figure 1A). However, there was no significant difference in ASMRs between females and males (AAPC among women: 11.24%; 95% CI, 10.09%-12.40%; *P* < .001; among men: 11.04%; 95% CI, 9.56%-12.63%; *P* < .001) as well as for projected values (Table 2 and Figure 1B).

ASMRs rose for all major racial ethnic groups, with the highest ASMR increase observed for the non-Hispanic White group (0.28 per 100 000 persons in 2006 to 1.43 per 100 000 persons in 2023; AAPC, 11.12%; 95% CI, 9.48%-12.83%; *P* < .001), followed by those of the Hispanic (0.28 to 1.32 per 100 000 persons; AAPC, 10.67%; 95% CI, 9.11%-12.26%; *P* < .001), non-Hispanic Black (0.19 to 0.61 per 100 000 persons; AAPC: 9.20%; 95% CI, 7.32%-11.11%; *P* < .001), and non-Hispanic Asian (0.15 to 0.39 per 100 000 persons; AAPC, 7.97%; 95% CI, 4.66%-11.75%; *P* < .001) groups, with increasing trends projected to 2040 for all these racial ethnic groups (Table 2 and Figure 1C). Among metropolitan categories, the highest rise was observed in nonmetropolitan areas (AAPC: 13.50%; 95% CI, 10.70%-16.32%; *P* < .001), followed by medium and small metropolitan areas, and the lowest rate in large central metropolitan areas (Table 2). Similar increased patterns of metropolitan categories were observed in the forecasted trends (Figure 1D). Consistent projected trends of MASLD-related mortality were observed using constructed linear regression model (eFigure 2 in Supplement 1).

### Subgroup Analysis for All-Cause MASLD-Related Mortality by Race and Ethnicity

#### By Age and Race and Ethnicity

**Figure 2.**
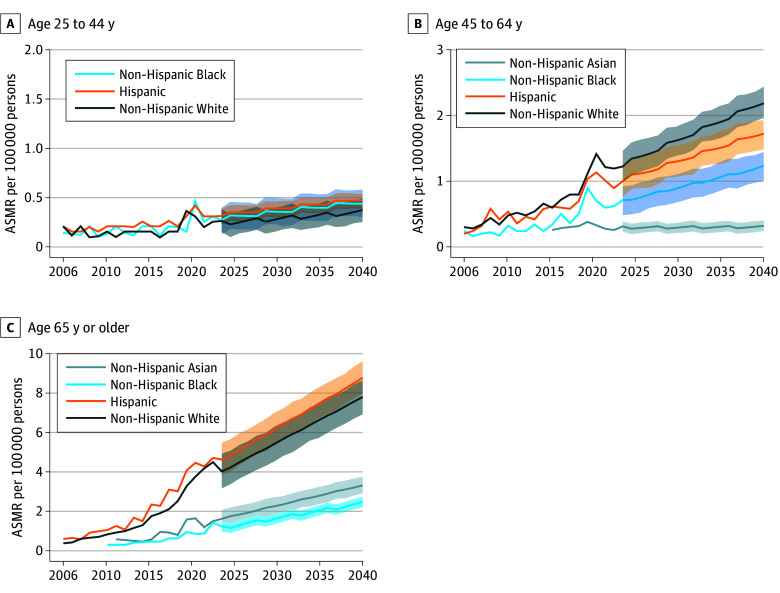
Age-Standardized Mortality Rates (ASMRs) and Projected Values for Metabolic Dysfunction–Associated Steatotic Liver Disease in the United States, 2006-2040, by Age and Race and Ethnicity There were very few metabolic dysfunction–associated steatotic liver disease–related deaths for Asian individuals aged 25 to 44 years recorded in the database. Accordingly, the data for Asian individuals in Figure 2A are not shown.

For those aged 25 to 44 years, there were no significant differences in the steady rise of ASMRs among Hispanic (AAPC, 4.13%; 2.30%-6.00%; *P* < .001), non-Hispanic Black (AAPC, 4.92%; 95% CI, 1.54%-8.53%; *P* = .006), and non-Hispanic White (AAPC, 3.61%; 95% CI, 0.11%-7.16%; *P* = .04) groups, with similar trends in the forecasted periods (Figure 2A; eTable 3 and eFigure 3A in Supplement 1). For those aged 45 to 64 years, there were modest ASMR increases from 2006 to 2023, with AAPCs of 8.49% (95% CI, 5.35%-11.63%), 8.86% (95% CI, 6.02%-11.81%), and 9.51% (95% CI, 7.91%-11.14%) in Hispanic, non-Hispanic Black, and non-Hispanic White groups, respectively (eTable 3 and eFigure 3B in Supplement 1). Similar increasing trends were observed through 2040 among these 3 racial ethnic groups (Figure 2B). Among those aged 65 years or older group, the steepest AAPC rise during 2006 to 2023 was for the non-Hispanic White group (15.56%; 95% CI, 14.46%-16.67%; *P* < .001), followed by those of the Hispanic (14.16%; 95% CI, 12.71%-15.69%; *P* < .001), non-Hispanic Black (12.98%; 95% CI, 8.56%-18.05%; *P* < .001), and non-Hispanic Asian (APC, 11.91%; 95% CI, 2.47%-23.82%) groups (eTable 3 and eFigure 3C in Supplement 1). The largest increase was projected for the Hispanic (4.63 to 8.77 per 100 000 persons) and non-Hispanic White (4.03 to 7.80 per 100 000 persons) groups through 2040 (Figure 2C).

#### By Sex and Race and Ethnicity

**Figure 3.**
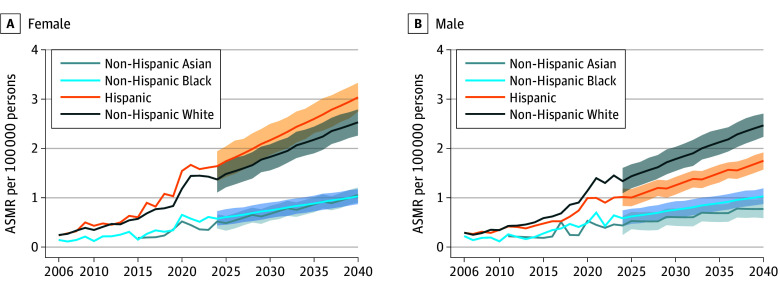
Age-Standardized Mortality Rates (ASMRs) and Projected Values for Metabolic Dysfunction–Associated Steatotic Liver Disease in the United States, 2006-2040, by Sex and Race and Ethnicity

Among women, the highest ASMR increase was observed for the Hispanic population (AAPC, 12.08%; 95% CI, 10.36% to 13.80%; *P* < .001), followed by those of the non-Hispanic White (AAPC, 11.16%; 95% CI, 9.80% to 12.55%; *P* < .001), non-Hispanic Asian (AAPC, 10.55%; 95% CI, 3.94% to 18.10%; *P* < .001), and non-Hispanic Black (AAPC, 9.81%; 95% CI, 6.92% to 12.83%; *P* < .001) groups (eTable 4 and eFigure 4A in Supplement 1). The largest increase was projected for the Hispanic and non-Hispanic White groups and plateaued for the non-Hispanic Asian and non-Hispanic Black groups (Figure 3A). Among men, the 2006 to 2023 AAPCs for ASMRs showed a significant increase for all racial and ethnic groups, with the highest change for the non-Hispanic White group (non-Hispanic White: AAPC, 11.22%; 95% CI, 9.90% to 12.57%; Hispanic: AAPC, 7.91%; 95% CI, 6.82% to 8.74%; non-Hispanic Asian: AAPC, 7.83%; 95% CI, −0.59% to 18.27%; non-Hispanic Black: AAPC, 7.65%; 95% CI, 4.96% to 11.14%) (eTable 4 and eFigure 4B in Supplement 1). Similar forecasted trends were observed for these 4 racial ethnic groups (Figure 3B).

#### By Urbanization and Race and Ethnicity

In large central metropolitan areas, a significant change in ASMR was observed mainly for the non-Hispanic White (AAPC, 10.73%; 95% CI, 8.88%-11.69%; *P* < .001) and Hispanic (AAPC, 10.48%; 95% CI, 8.67%-12.36%; *P* < .001) groups, while it plateaued for the non-Hispanic Asian (AAPC, 8.52%; 95% CI, 2.41%-18.44%; *P* = .008) and non-Hispanic Black (AAPC, 7.00%; 95% CI, 2.60%-11.52%; *P* < .001) groups, with similar trends forecasted to 2040 (eTable 5 and eFigures 5A and 6A in Supplement 1). In medium and small metropolitan areas, the highest ASMR increase was observed for the Hispanic (AAPC, 13.19%; 95% CI, 8.73%-17.85%; *P* < .001) and non-Hispanic White (AAPC, 10.68%; 95% CI, 9.49%-11.90%; *P* < .001) groups, with the lowest change observed for the non-Hispanic Black group (AAPC, 6.66%; 95% CI, 0.20%-13.91%; *P* = .04) (eTable 5 and eFigures 5B and 6B in Supplement 1). In nonmetropolitan areas, there was similar increasing pattern for the Hispanic and non-Hispanic White groups (Hispanic: AAPC, 13.37% [95% CI, 5.96%-21.48%]; non-Hispanic White: AAPC, 13.77% [95% CI, 11.20%-16.31%]), but the projected rates were higher in the Hispanic than the non-Hispanic White group (eTable 5 and eFigures 5C and 6C in Supplement 1).

## Discussion

In this population-based study, the MASLD-related mortality rate increased rapidly during 2006 to 2023 and was projected to rise to 2040. This upward trend was more prominent among older adults (aged ≥65 years), but no sex difference was observed during this period. Racial and ethnic disparities were also evident, with non-Hispanic White populations, followed by Hispanic, non-Hispanic Black, and non-Hispanic Asian populations, experiencing a rising trend. Among metropolitan categories, the highest increase was observed in nonmetropolitan areas. In subgroup analysis by race and ethnicity, there were marked disparities among different age, sex, and urbanization categories.

In a recent population-based US study, MASLD-related mortality rose from an age-adjusted mortality rate of 0.2 to 1.7 per 100 000 persons, with an AAPC of 10.0% between 1999 and 2022.^3^ Another modeling study suggested that among the MASLD population in the United States, 28 200 MASLD-related deaths occurred in 2015, and there were 78 300 projected deaths in 2030.^18^ Our study reinforced findings from prior studies showing that the MASLD-related mortality rate increased from 0.25 per 100 000 persons in 2006 to 1.27 per 100 000 persons in 2023, with an AAPC of 10.21%, indicating forecasted data to 2.24 per 100 000 persons in 2040.^1,19,20,21^ As the population ages and demographic characteristics shift, the higher burden of metabolic diseases may increase the risk of MASLD and its progression to advanced liver disease, potentially contributing to higher MASLD mortality rates.^22^ The increase of MASLD mortality rates from 2018 to 2021 can be attributed to the increased burden of metabolic diseases and the underdiagnosis of MASLD due to lack of widespread awareness.^23,24^ However, after 2021, advances in MASLD management, early detection, and intervention may help reverse the projected increasing mortality trend.^25^

Paik et al^13^ reported that the age-specific all-cause death rate among individuals with MASLD in the United States was the highest for decedents aged 75 years or older, followed by those aged 65 to 74, 55 to 64, 45 to 54, and 20 to 44 years.^13^ In a large cohort of patients with MASLD from Hong Kong, patients aged 70 to 79 and 80 to 90 years had 5.1% and 5.9% liver-related mortality, compared with less than 3% in groups younger than 70 years.^6^ We found that the group with ages starting at 65 years had steeper upward mortality trends when compared with middle- and younger-aged groups. The disparities between age groups emphasize the importance of targeted public health strategies for older populations.

Additionally, we provided data to help clarify whether sex-based differences in MASLD-related mortality rate are significant. Some studies have demonstrated that the mortality rate is increasing significantly more in women than men among adults with MASLD.^3,13,26^ However, other studies have suggested that no sex difference is observed for all-cause mortality associated with MASLD.^11^ Our study demonstrated that the mortality rate of MASLD was similar between males and females, but further attention still should be directed toward promoting sex equality in the diagnosis and treatment of MASLD.

Our study also found widened disparities of MASLD-related mortality rate among the different racial and ethnic groups. Hispanic and non-Hispanic White people had highest mortality rates and the steepest upward trend during 2006 to 2023 and forecast to 2040. Additionally, Hispanic women had the highest mortality rate increase, followed by non-Hispanic White women, with the opposite direction among non-Hispanic White men, who had higher rates than Hispanic men. This may be due to a genetic predisposition as well as higher rates of obesity and diabetes.^27,28^ Hispanic women may also experience more socioeconomic and cultural challenges than men that exacerbate their health outcomes.^29^ Our data differed from another study from Ilyas et al^3^ using the NVSS dataset between 1999 and 2022, which showed that non-Hispanic White patients had the highest mortality rate, but African American patients showed a not statistically significant change during this time period.^3^ The study from Ilyas et al^3^ was based on the population with nonalcoholic fatty liver disease, which did not require inclusion of cardiometabolic factors as the old diagnostic criteria,^30^ while our current study reflected the data for MASLD population with its updated diagnostic criteria that requires presence of at least 1 cardiometabolic risk factor.^16^ The discrepant definition of these 2 cohorts may lead to the different mortality trends of specific racial and ethnic groups. Another study reported higher overall mortality rates among Black patients compared with Asian, Hispanic, and White patients^31^; however, this study examined mortality of a cohort presenting to a university medical center, which can be subjected to selection bias, while our data included more generalizable data for more than 99% of decedents in the United States.

Regarding differences by population density, data examining the epidemiology of MASLD in the rural population are limited, although changes in lifestyle, diet profiles, and metabolic profiles of the rural population have been observed for the last few decades.^32,33^ Our findings filled in this gap by demonstrating that nonmetropolitan areas had the highest rise, followed by medium and small metropolitan areas, with the lowest in large metropolitan areas. People living in rural areas tend to have additional barriers to care, which may contribute to the higher burden of undiagnosed or untreated MASLD.^34^ Higher levels of physical inactivity and unhealthy dietary habits among rural residents may increase the prevalence of risk factors such as obesity and insulin resistance, which are closely associated with MASLD development.^35,36^ Together, these data are important to inform policy and resource allocation, which should be directed toward rural areas.

### Limitations

We acknowledge several limitations to this study. First, we relied on *ICD-10* codes to identify MASLD, but they can be miscoded, which may include misclassification of patients who have alcoholic steatosis, leading to either an undercoding or overcoding of the data we used. However, previous study reported a high positive predictive value for *ICD-10* codes to identify MASLD; it was 0.91 (95% CI, 0.82-0.97).^37^ Second, the number of reported deaths related to MASLD may be underestimated due to a number of factors. MASLD is severely underdiagnosed, and awareness of MASLD in the general population was less than 10%.^38^ As a result, many MASLD-related deaths may not be attributed to MASLD. MASLD often coexists with other conditions, such as cardiovascular diseases, which are more commonly recognized as the contributing causes of death. Additionally, cardiometabolic disorders may be underreported on death certificates as contributing factors, even if they actually contributed to death. Furthermore, there is potential collinearity among multiple demographic factors, which might influence mortality. However, we cannot adjust for these demographic factors in multivariate models because no individual decedent demographic data can be obtained due to patient privacy policy. Accordingly, we provided detailed subgroup analyses stratified by demographic factors to investigate the association of each factor with the MASLD mortality rate. Furthermore, actual future mortality may also differ from projected values if there are major changes that influence the prevalence or natural history of MASLD, such as major therapeutic advances that can prevent the progression of MASLD.

## Conclusions

This study found that MASLD-related mortality increased rapidly from 2006 to 2023, and it was predicted to continue to increase over the next 20 years, with the largest disparities among those aged 65 years and older, Hispanic and non-Hispanic White populations, and nonmetropolitan populations. The findings provide clinical recommendations on these high-risk populations for MASLD screening to detect MASLD development early. Early intervention with lifestyle modifications and medications for high-risk groups could help prevent the development of MASLD. Targeted awareness campaigns can help inform high-risk groups on lifestyle factors that contribute to MASLD, such as diet, physical activity, and alcohol consumption.

## Notes

Supplement 1.
eMethods.
**eTable 1.** Root Mean Square Error (RMSE) Comparison Between Prophet and Constructed Linear Regression Model Across Different Groups **eTable 2. **Proportion of Deaths Related to Liver Transplant Among Decedents With MASLD as a Contributing Cause of Death in the United States, 2006-2023 **eTable 3.** Annual Percentage Change and Age-Standardized Mortality Rate Among Decedents With MASLD as a Contributing Cause of Death, Stratified by Age (25-44, 45-64, and ≥65 years) Plus Race and Ethnicity (Non-Hispanic Asian, Black, Hispanic and Non-Hispanic White), Between 2006 and 2023, Estimated by Joinpoint Regression Analysis **eTable 4.** Annual Percentage Change and Age-Standardized Mortality Rate Among Decedents With MASLD as a Contributing Cause of Death, Stratified by Sex (Female and Male) Plus Race and Ethnicity (Non-Hispanic Asian, Black, Hispanic and Non-Hispanic White), Between 2006 and 2023, Estimated by Joinpoint Regression Analysis **eTable 5.** Annual Percentage Change and Age-Standardized Mortality Rate Among Decedents With MASLD as a Contributing Cause of Death, Stratified by Urbanization (Large and Fringe Metropolitan, Medium and Small Metropolitan, and Nonmetropolitan) Plus Race and Ethnicity (Non-Hispanic Asian, Black, Hispanic, and Non-Hispanic White), Between 2006 and 2020, Estimated by Joinpoint Regression Analysis **eFigure 1.** Annual Percentage Change and Age-Standardized Mortality Rate Among Decedents With MASLD as a Contributing Cause of Death Between 2006 and 2023, (A) Overall and by Age, (B) Sex, (C) Race and Ethnicity and (D) Urbanization, Estimated by Joinpoint Regression Analysis **eFigure 2. **Age-Standardized Mortality Rates (ASMRs) and Projected Values for MASLD in the United States in 2006-2040, (A) Overall and by Age, (B) Sex, (C) Race and Ethnicity, and (D) Urbanization, Estimated by Constructed Linear Regression Model **eFigure 3. **Annual Percentage Change and Age-Standardized Mortality Rate Among Decedents With MASLD as a Contributing Cause of Death, Stratified by (A) Age 25-44, (B) Age 45-64, and (C) Age ≥65 Years Plus Race and Ethnicity (Non-Hispanic Asian, Black, Hispanic, and Non-Hispanic White) Between 2006 and 2023, Estimated by Joinpoint Regression Analysis **eFigure 4. **Annual Percentage Change and Age-Standardized Mortality Rate Among Decedents With MASLD as a Contributing Cause of Death, Stratified by Sex (A) Female and (B) Male Plus Race and Ethnicity (Non-Hispanic Asian, Black, Hispanic, and Non-Hispanic White) Between 2006 and 2023, Estimated by Joinpoint Regression Analysis **eFigure 5. **Annual Percentage Change and Age-Standardized Mortality Rate Among Decedents With MASLD as a Contributing Cause of Death, Stratified by Urbanization (A) Large and Fringe Metropolitan, (B) Medium and Small Metropolitan, and (C) Nonmetropolitan Plus Race and Ethnicity (Non-Hispanic Asian, Black, Hispanic, and Non-Hispanic White) Between 2006 and 2020, Estimated by Joinpoint Regression Analysis **eFigure 6.** Age-Standardized Mortality Rates (ASMRs) and Projected Values for MASLD in the United States in 2006-2040, Stratified by Urbanization (A) Large and Fringe Metropolitan, (B) Medium and Small Metropolitan, and (C) Nonmetropolitan Plus Race and Ethnicity (Non-Hispanic Asian, Black, Hispanic, and Non-Hispanic White)

Supplement 2.
Data Sharing Statement
