# Supplementary material for: Projected Trends in Metabolic Dysfunction–Associated Steatotic Liver Disease Mortality Through 2040
Source: JAMA Netw Open. 2025 Jun 17;8(6):e2516367. doi: 10.1001/jamanetworkopen.2025.16367 (PMC12175021; doi:10.1001/jamanetworkopen.2025.16367)
Supplement: Supplement 1. — eMethods. eTable 1. Root Mean Square Error (RMSE) Comparison Between Prophet and Constructed Linear Regression Model Across Different Groups eTable 2. Proportion of Deaths Related to Liver Transplant Among Decedents With MASLD as a Contributing Cause of Death in the United States, 2006-2023 eTable 3. Annual Percentage Change and Age-Standardized Mortality Rate Among Decedents With MASLD as a Contributing Cause of Death, Stratified by Age (25-44, 45-64, and ≥65 years) Plus Race and Ethnicity (Non-Hispanic Asian, Black, Hispanic and Non-Hispanic White), Between 2006 and 2023, Estimated by Joinpoint Regression Analysis eTable 4. Annual Percentage Change and Age-Standardized Mortality Rate Among Decedents With MASLD as a Contributing Cause of Death, Stratified by Sex (Female and Male) Plus Race and Ethnicity (Non-Hispanic Asian, Black, Hispanic and Non-Hispanic White), Between 2006 and 2023, Estimated by Joinpoint Regression Analysis eTable 5. Annual Percentage Change and Age-Standardized Mortality Rate Among Decedents With MASLD as a Contributing Cause of Death, Stratified by Urbanization (Large and Fringe Metropolitan, Medium and Small Metropolitan, and Nonmetropolitan) Plus Race and Ethnicity (Non-Hispanic Asian, Black, Hispanic, and Non-Hispanic White), Between 2006 and 2020, Estimated by Joinpoint Regression Analysis eFigure 1. Annual Percentage Change and Age-Standardized Mortality Rate Among Decedents With MASLD as a Contributing Cause of Death Between 2006 and 2023, (A) Overall and by Age, (B) Sex, (C) Race and Ethnicity and (D) Urbanization, Estimated by Joinpoint Regression Analysis eFigure 2. Age-Standardized Mortality Rates (ASMRs) and Projected Values for MASLD in the United States in 2006-2040, (A) Overall and by Age, (B) Sex, (C) Race and Ethnicity, and (D) Urbanization, Estimated by Constructed Linear Regression Model eFigure 3. Annual Percentage Change and Age-Standardized Mortality Rate Among Decedents With MASLD as a Contributing Cause of Death, Strat [file jamanetwopen-e2516367-s001.pdf]

Supplemental Online Content

Zhang X, Linden S, Levesley CR, et al. Projected trends in metabolic dysfunction–associated steatotic liver disease mortality through 2040. *JAMA Netw Open.* 2025;8(6):e2516367. doi:10.1001/jamanetworkopen.2025.16367

eMethods.

eTable 1. Root Mean Square Error (RMSE) Comparison Between Prophet and Constructed Linear Regression Model Across Different Groups

eTable 2. Proportion of Deaths Related to Liver Transplant Among Decedents With MASLD as a Contributing Cause of Death in the United States, 2006-2023

eTable 3. Annual Percentage Change and Age-Standardized Mortality Rate Among Decedents With MASLD as a Contributing Cause of Death, Stratified by Age (25-44, 45-64, and ≥65 years) Plus Race and Ethnicity (Non-Hispanic Asian, Black, Hispanic and Non-Hispanic White), Between 2006 and 2023, Estimated by Joinpoint Regression Analysis

eTable 4. Annual Percentage Change and Age-Standardized Mortality Rate Among Decedents With MASLD as a Contributing Cause of Death, Stratified by Sex (Female and Male) Plus Race and Ethnicity (Non-Hispanic Asian, Black, Hispanic and Non-Hispanic White), Between 2006 and 2023, Estimated by Joinpoint Regression Analysis

eTable 5. Annual Percentage Change and Age-Standardized Mortality Rate Among Decedents With MASLD as a Contributing Cause of Death, Stratified by Urbanization (Large and Fringe Metropolitan, Medium and Small Metropolitan, and Nonmetropolitan) Plus Race and Ethnicity (Non-Hispanic Asian, Black, Hispanic, and Non-Hispanic White), Between 2006 and 2020, Estimated by Joinpoint Regression Analysis

eFigure 1. Annual Percentage Change and Age-Standardized Mortality Rate Among Decedents With MASLD as a Contributing Cause of Death Between 2006 and 2023, (A) Overall and by Age, (B) Sex, (C) Race and Ethnicity and (D) Urbanization, Estimated by Joinpoint Regression Analysis

eFigure 2. Age-Standardized Mortality Rates (ASMRs) and Projected Values for MASLD in the United States in 2006-2040, (A) Overall and by Age, (B) Sex, (C) Race and Ethnicity, and (D) Urbanization, Estimated by Constructed Linear Regression Model

eFigure 3. Annual Percentage Change and Age-Standardized Mortality Rate Among Decedents With MASLD as a Contributing Cause of Death, Stratified by (A) Age 25-44, (B) Age 45-64, and (C) Age ≥65 Years Plus Race and Ethnicity (Non-Hispanic Asian,

© 2025 Zhang X et al. *JAMA Network Open*.

Black, Hispanic, and Non-Hispanic White) Between 2006 and 2023, Estimated by Joinpoint Regression Analysis

eFigure 4. Annual Percentage Change and Age-Standardized Mortality Rate Among Decedents With MASLD as a Contributing Cause of Death, Stratified by Sex (A) Female and (B) Male Plus Race and Ethnicity (Non-Hispanic Asian, Black, Hispanic, and Non-Hispanic White) Between 2006 and 2023, Estimated by Joinpoint Regression Analysis

eFigure 5. Annual Percentage Change and Age-Standardized Mortality Rate Among Decedents With MASLD as a Contributing Cause of Death, Stratified by Urbanization (A) Large and Fringe Metropolitan, (B) Medium and Small Metropolitan, and (C) Nonmetropolitan Plus Race and Ethnicity (Non-Hispanic Asian, Black, Hispanic, and Non-Hispanic White) Between 2006 and 2020, Estimated by Joinpoint Regression Analysis

eFigure 6. Age-Standardized Mortality Rates (ASMRs) and Projected Values for MASLD in the United States in 2006-2040, Stratified by Urbanization (A) Large and Fringe Metropolitan, (B) Medium and Small Metropolitan, and (C) Nonmetropolitan Plus Race and Ethnicity (Non-Hispanic Asian, Black, Hispanic, and Non-Hispanic White)

This supplemental material has been provided by the authors to give readers additional information about their work.

© 2025 Zhang X et al. *JAMA Network Open*.

eMethods

Age-standardized mortality rates

Age-standardized mortality rates were developed using the age structure (25-85+ years) from the 2000 USA Census Standard Population and the direct standardization method (by multiplying the age-specific mortality rates of the study population to the number of persons in each age group of the standard population), but no other factors such as sex, race and ethnicity, and urbanization, were adjusted for in the analysis.

Joinpoint regression analysis

To determine the nationwide trend of MASLD-related mortality, we conducted joinpoint regression analysis which typically selects the optimal number of joinpoints using permutation test method[1]. The annual percentage change (APC) is estimated for each segment (defined by the joinpoints) using piecewise linear regression models, and the change in trend at each joinpoint is captured by the change in the slope of the log-linear segments.

Prophet prediction model

Prophet is an open-source time series prediction statistical model developed by the Facebook team. Prophet model decomposes time series data into multiple modules such as trend items, seasonal items, and holiday effects based on an additive model[2]. After fitting each module, the predicted values of each module are combined based on the generalized additive model to obtain the prediction results of future time series, and provide a confidence interval for each predicted value to evaluate the reliability of the prediction results.

Its algorithm model can be simply expressed as: $y(t) = g(t) + s(t) + h(t) + \varepsilon t$; Here, $g(t)$ represents the trend term of the time series, $s(t)$ represents the seasonal term, $h(t)$ represents the holiday term, and εt represents the error term, which is usually assumed to follow a normal distribution. This is an additive form based on the decomposition method. Another form is a multiplicative form based on the decomposition method, which can be expressed as $y(t) = g(t) \times s(t) \times h(t) \times \varepsilon t$; Simply take the logarithm of the variables on both sides to convert the multiplicative form to the additive form.

The selection of Prophet model was based on the distribution of the data and model fitness which assessed through the root mean squared error (RMSE). RMSE measures the average magnitude of the errors between predicted and actual values, which can assess the predictive accuracy of the model. Lower RMSE means the forecast is more reliable. RMSE is defined as:

$$RMSE = \sqrt{\frac{1}{n}\sum_{i=1}^{n}(\hat{y}_i - y_i)^2}$$

© 2025 Zhang X et al. *JAMA Network Open*.

Compared with other time series models, Prophet model stands out for its flexibility, ease of use, and robustness in handling various complexities such as seasonality, holidays, missing data, outliers, and trend changes[2]. It simplifies the forecasting process while maintaining high predictive accuracy, making it a favored choice for time series forecasting, especially when dealing with non-linear trends, irregular time intervals, non-stationary data, and other potential impact by external factors.

eReferences

1. Kim HJ, Fay MP, Feuer EJ, Midthune DN. Permutation tests for joinpoint regression with applications to cancer rates. Stat Med. Feb 15 2000;19(3):335-51.

2.Taylor SJ, Letham B. Forecasting at scale. The American Statistician, 2018, 72(1): 37-45.

© 2025 Zhang X et al. *JAMA Network Open*.

eTable 1. Root Mean Square Error (RMSE) Comparison Between Prophet and Constructed Linear Regression Model Across Different Groups

Group	RMSE*	
	Prophet model	Constructed linear regression model
**Overall**	0.118	0.100
**Age**		
**25-44 years**	0.040	0.043
**45-64 years**	0.112	0.119
**≥65 years**	0.375	0.398
**Sex**		
**Female**	0.117	0.125
**Male**	0.110	0.116
**Race and ethnicity**		
**Non-Hispanic Asian**	0.088	0.066
**Non-Hispanic Black**	0.066	0.070
**Hispanic**	0.131	0.139
**Non-Hispanic White**	0.133	0.141
**Urbanization**		
**Large and fringe metro**	0.035	0.126
**Medium and small metro**	0.042	0.039
**Non-metro**	0.091	0.046

*RMSE measures the average magnitude of the errors between predicted and actual values, which can assess the predictive accuracy of the model. Lower RMSE means the forecast is more reliable. RMSE, Root Mean Square Error; metro, metropolitan

© 2025 Zhang X et al. *JAMA Network Open*.

eTable 2. Proportion of Deaths Related to Liver Transplant Among Decedents With MASLD as a Contributing Cause of Death in the United States, 2006-2023

	Deaths [%] 2006-2023	Deaths [%] 2006	Deaths [%] 2010	Deaths [%] 2015	Deaths [%] 2020	Deaths [%] 2021	Deaths [%] 2022	Deaths [%] 2023
**Overall**	27,961 [100.0]	491 [100.0]	657 [100.0]	1,269 [100.0]	2,885 [100.0]	3,319 [100.0]	3,314 [100.0]	3,464 [100.0]
**Liver transplant**	575 [2.1]	22 [4.5]	34 [5.2]	27 [2.1]	36 [1.2]	35 [1.1]	20 [0.6]	50 [1.4]

© 2025 Zhang X et al. *JAMA Network Open*.

eTable 3. Annual Percentage Change and Age-Standardized Mortality Rate Among Decedents With MASLD as a Contributing Cause of Death, Stratified by Age (25-44, 45-64, and ⩾65 years) Plus Race and Ethnicity (Non-Hispanic Asian, Black, Hispanic and Non-Hispanic White), Between 2006 and 2023, Estimated by Joinpoint Regression Analysis

	**Average APC (95% CI)**	***P* value**	**Trend segment**		***P* value**
	**2006-2023**		**Year**	**APC (95% CI)**	
**Age**					
**25-44 years**					
Non-Hispanic Black	4.92 (1.54-8.53)	0.006	2006-2023	4.92 (1.54-8.53)	0.006
Hispanic	4.13 (2.30-6.00)	<0.001	2006-2023	4.13 (2.30-6.00)	<0.001
Non-Hispanic White	3.61 (0.11-7.16)	0.042	2006-2023	3.61 (0.11-7.16)	0.042
**45-64 years**					
Non-Hispanic Black	8.86 (6.02-11.81)	<0.001	2006-2023	8.86 (6.02-11.81)	<0.001
Hispanic	8.49 (5.35-11.63)	<0.001	2006-2023	8.49 (5.35-11.63)	<0.001
Non-Hispanic White	9.51 (7.91-11.14)	<0.001	2006-2023	9.51 (7.91-11.14)	<0.001
**⩾65 years**					
Non-Hispanic Asian	11.91 (2.47-23.82)	0.016	2012-2023	11.91 (2.47-23.82)	0.016
Non-Hispanic Black	12.98 (8.56-18.05)	<0.001	2011-2023	12.98 (8.56-18.05)	<0.001
Hispanic	14.16 (12.71-15.69)	<0.001	2006-2023	14.16 (12.71-15.69)	<0.001
Non-Hispanic White	15.56 (14.46-16.67)	<0.001	2006-2023	15.56 (14.46-16.67)	<0.001

APC was calculated by joinpoint regression analysis. Due to very few MASLD-related deaths recorded in non-Hispanic Asian population among age 25-44 years and 45-64 years, APC of non-Hispanic Asian population in these two categories were not shown in the Table. APC, annual percentage change; CI, confidence interval

© 2025 Zhang X et al. *JAMA Network Open*.

eTable 4. Annual Percentage Change and Age-Standardized Mortality Rate Among Decedents With MASLD as a Contributing Cause of Death, Stratified by Sex (Female and Male) Plus Race and Ethnicity (Non-Hispanic Asian, Black, Hispanic and Non-Hispanic White), Between 2006 and 2023, Estimated by Joinpoint Regression Analysis

	Average APC (95% CI)	*P* value	Trend segment		*P* value
	2006-2023		Year	APC (95% CI)	
**Sex**					
**Female**					
Non-Hispanic Asian	10.55 (3.94-18.10)	<0.001	2015-2020<br>2020-2023	23.31 (15.00-54.27)<br>-7.85 (-32.78 to 6.88)	0.001<br>0.344
Non-Hispanic Black	9.81 (6.92-12.83)	<0.001	2006-2023	9.81 (6.92-12.83)	<0.001
Hispanic	12.08 (10.36-13.80)	<0.001	2006-2023	12.08 (10.36-13.80)	<0.001
Non-Hispanic White	11.16 (9.80-12.55)	<0.001	2006-2023	11.16 (9.80-12.55)	<0.001
**Male**					
Non-Hispanic Asian	7.83 (-0.59 to 18.27)	0.066	2011-2023	7.83 (-0.59 to 18.27)	0.066
Non-Hispanic Black	7.65 (4.96-11.14)	<0.001	2006-2010<br>2010-2023	-5.33 (-28.52 to 9.05)<br>11.99 (8.31-31.05)	0.537<br>0.028
Hispanic	7.91 (6.82-8.74)	<0.001	2006-2017<br>2017-2020<br>2020-2023	6.36 (4.28-7.72)<br>21.81 (12.30-26.90)<br>0.76 (-10.41 to 7.32)	0.004<br>0.003<br>0.957
Non-Hispanic White	11.22 (9.90-12.57)	<0.001	2006-2014<br>2014-2023	8.83 (-1.15 to 11.98)<br>13.39 (8.13-24.64)	0.073<br>0.018

APC was calculated by joinpoint regression analysis. APC, annual percentage change; CI, confidence interval

© 2025 Zhang X et al. *JAMA Network Open*.

eTable 5. Annual Percentage Change and Age-Standardized Mortality Rate Among Decedents With MASLD as a Contributing Cause of Death, Stratified by Urbanization (Large and Fringe Metropolitan, Medium and Small Metropolitan, and Nonmetropolitan) Plus Race and Ethnicity (Non-Hispanic Asian, Black, Hispanic, and Non-Hispanic White), Between 2006 and 2020, Estimated by Joinpoint Regression Analysis

	**Average APC (95% CI)**	***P* value**	**Trend segment**		***P* value**
	**2006-2023**		**Year**	**APC (95% CI)**	
**Urbanization**					
**Large and fringe metro**					
Non-Hispanic Asian	8.52 (2.41-18.44)	0.008	2011-2013<br>2013-2020	-18.30 (-38.48 to 18.92)<br>17.69 (-12.65 to 62.13)	0.462<br>0.087
Non-Hispanic Black	7.00 (2.60-11.52)	<0.001	2006-2020	7.00 (2.60-11.52)	<0.001
Hispanic	10.48 (8.67-12.36)	<0.001	2006-2020	10.48 (8.67-12.36)	<0.001
Non-Hispanic White	10.73 (8.88-11.69)	<0.001	2006-2018<br>2018-2020	8.78 (2.97-11.79)<br>23.23 (9.17-30.89)	0.019<br><0.001
**Medium and small metro**					
Non-Hispanic Black	6.66 (0.20-13.91)	0.043	2006-2020	6.66 (0.20-13.91)	0.043
Hispanic	13.19 (8.73-17.85)	<0.001	2006-2020	13.19 (8.73-17.85)	<0.001
Non-Hispanic White	10.68 (9.49-11.90)	<0.001	2006-2020	10.68 (9.49-11.90)	<0.001
**Non-metro**					
Hispanic	13.37 (5.96-21.48)	<0.001	2012-2016<br>2016-2020	-3.22 (-34.88 to 17.48)<br>32.79 (10.77-95.92)	0.496<br>0.025
Non-Hispanic White	13.77 (11.20-16.31)	<0.001	2006-2020	13.77 (11.20-16.31)	<0.001

APC was calculated by joinpoint regression analysis. Due to very few MASLD-related deaths recorded in non-Hispanic Asian population among medium and small metro and non-metro categories, and very few deaths also recorded in non-Hispanic Black population among non-metro category, APC of non-Hispanic Asian and non-Hispanic Black populations in some categories were not shown in the Table. APC, annual percentage change; CI, confidence interval; metro, metropolitan

© 2025 Zhang X et al. *JAMA Network Open*.

eFigure 1. Annual Percentage Change and Age-Standardized Mortality Rate Among Decedents With MASLD as a Contributing Cause of Death Between 2006 and 2023, (A) Overall and by Age, (B) Sex, (C) Race and Ethnicity and (D) Urbanization, Estimated by Joinpoint Regression Analysis

Metro, metropolitan

(A) Overall and age

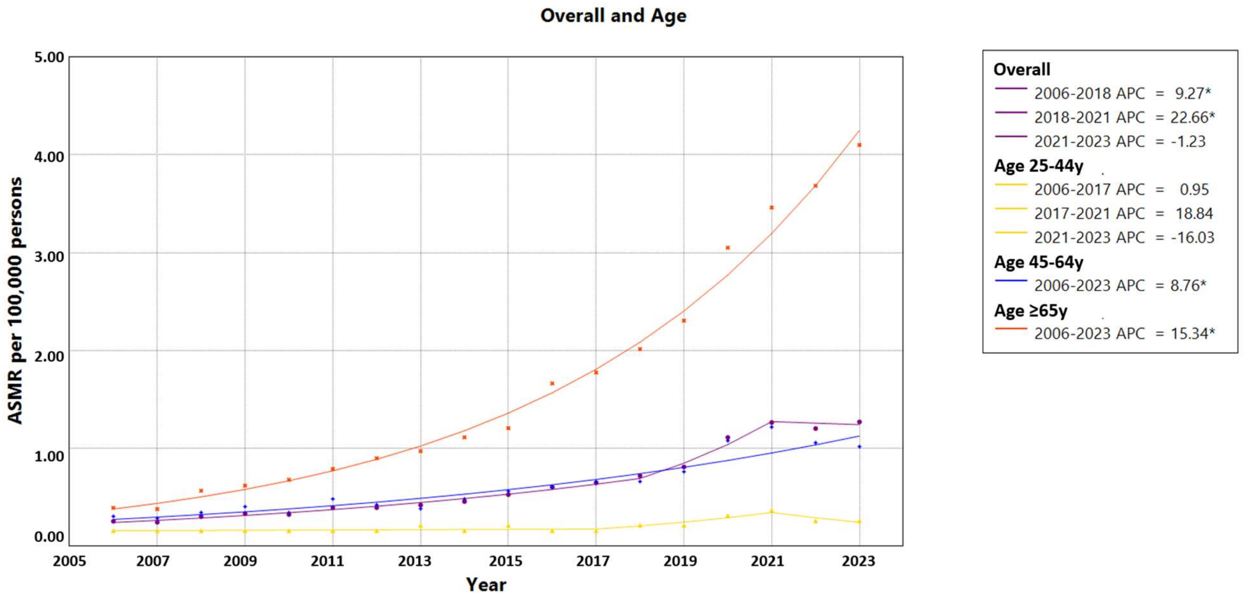

(B) Sex

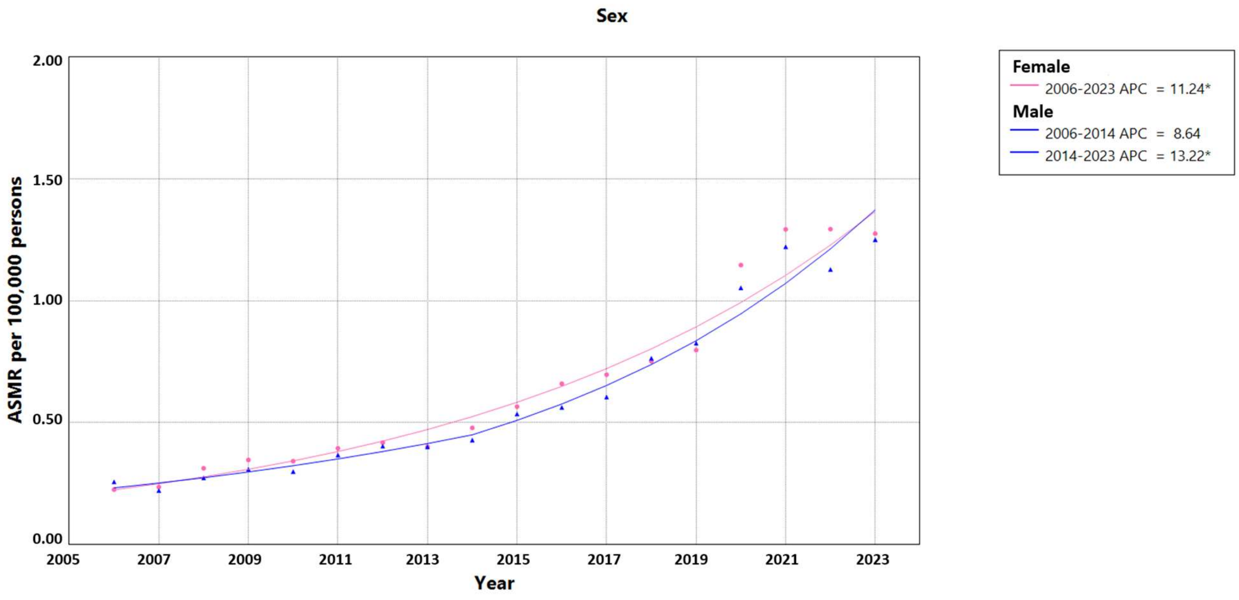

© 2025 Zhang X et al. *JAMA Network Open*.

(C) Race and ethnicity

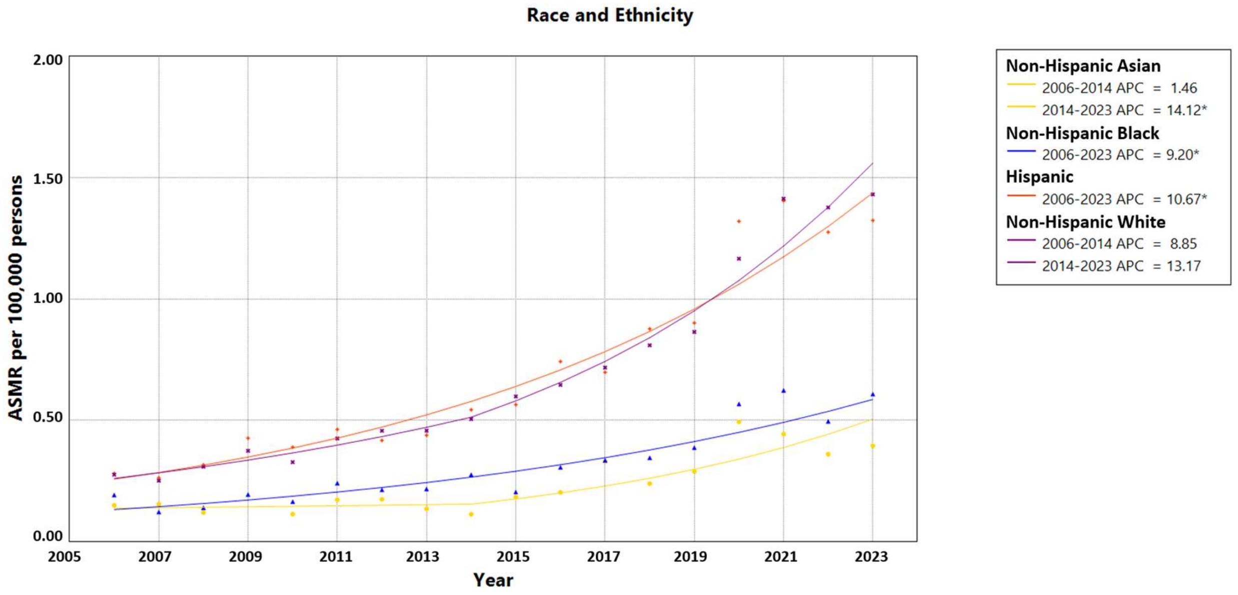

(D) Urbanization

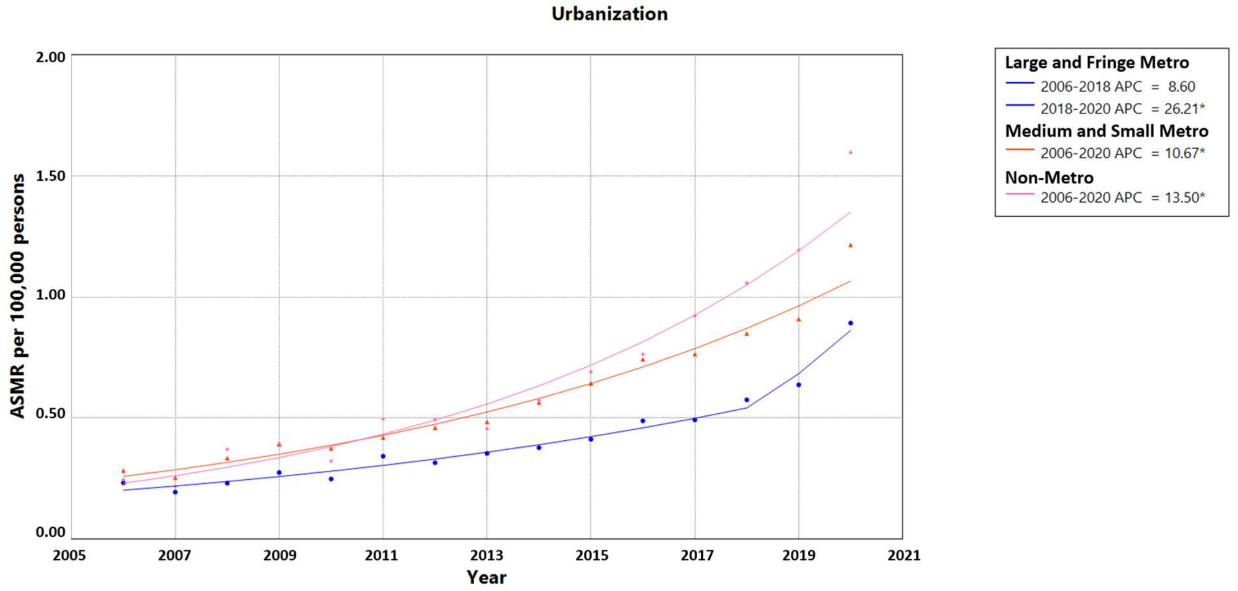

© 2025 Zhang X et al. *JAMA Network Open*.

eFigure 2. Age-Standardized Mortality Rates (ASMRs) and Projected Values for MASLD in the United States in 2006-2040, (A) Overall and by Age, (B) Sex, (C) Race and Ethnicity, and (D) Urbanization, Estimated by Constructed Linear Regression Model

Metro, metropolitan

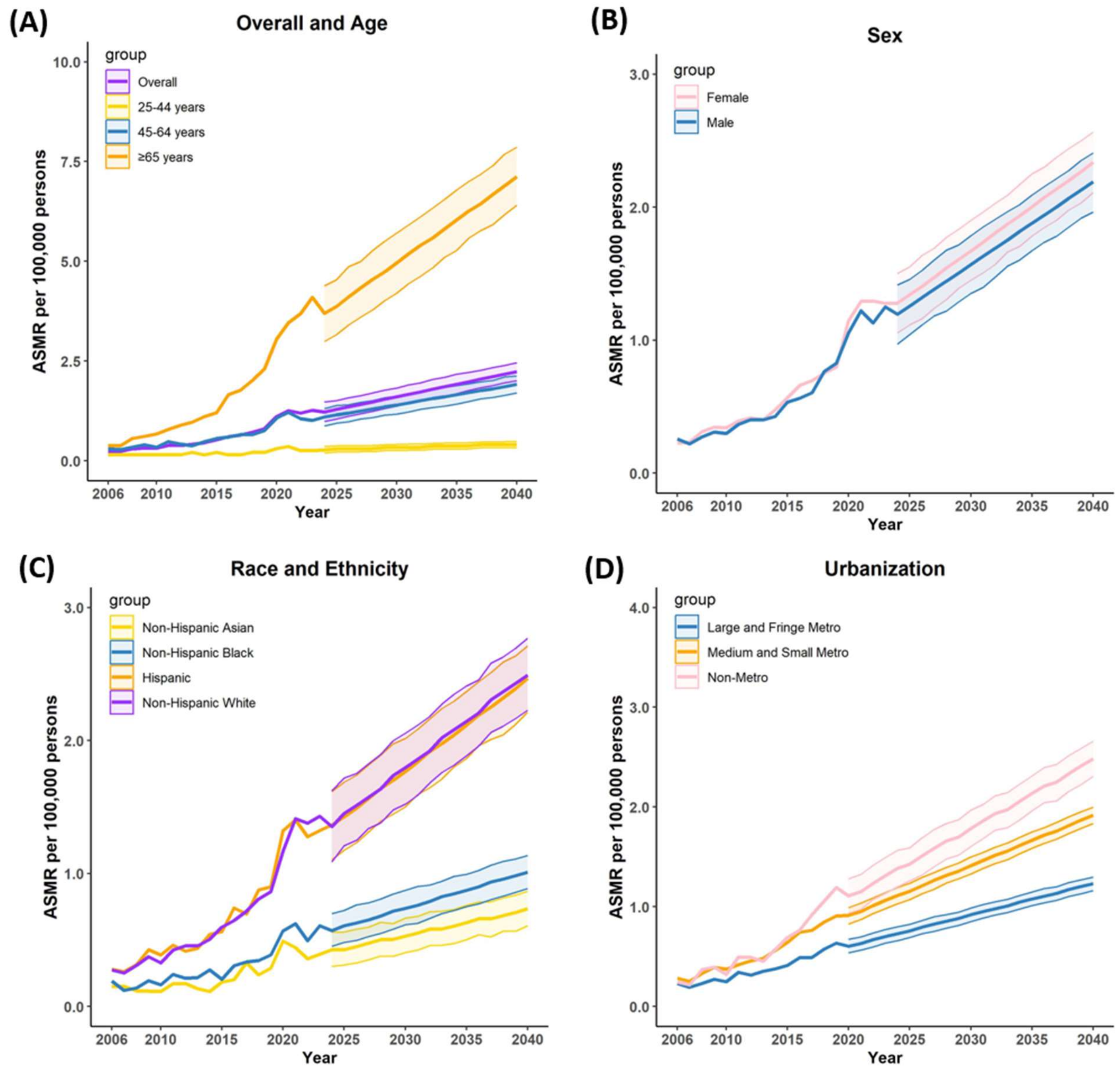

© 2025 Zhang X et al. *JAMA Network Open*.

eFigure 3. Annual Percentage Change and Age-Standardized Mortality Rate Among Decedents With MASLD as a Contributing Cause of Death, Stratified by (A) Age 25-44, (B) Age 45-64, and (C) Age ⩾ 65 Years Plus Race and Ethnicity (Non-Hispanic Asian, Black, Hispanic, and Non-Hispanic White) Between 2006 and 2023, Estimated by Joinpoint Regression Analysis

(A)

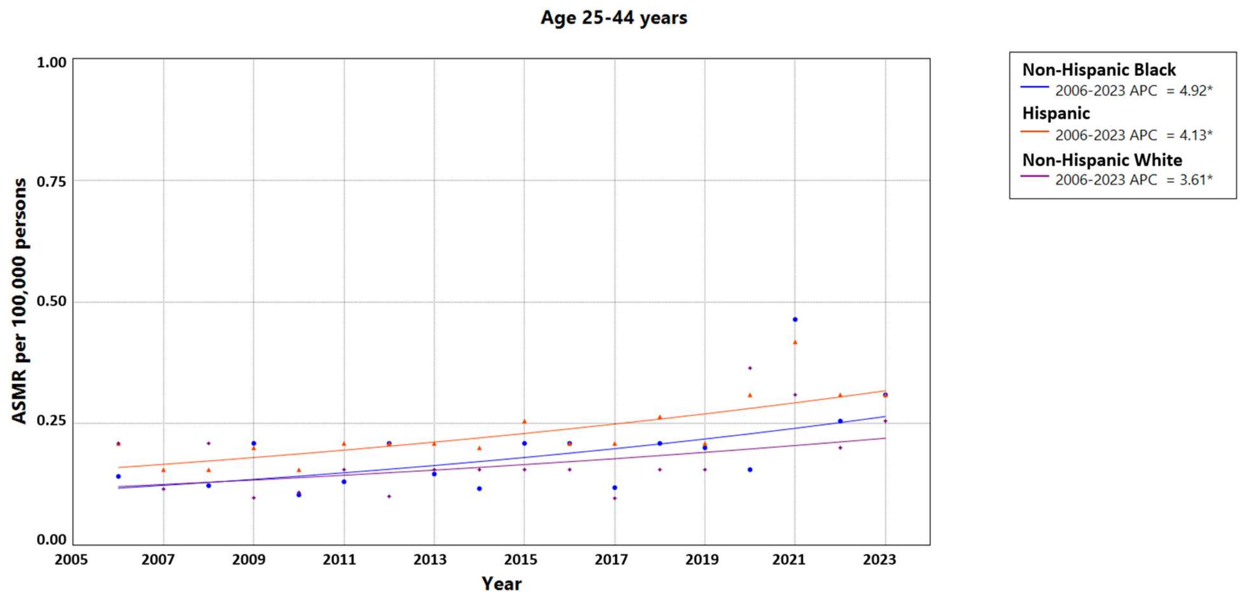

(B)

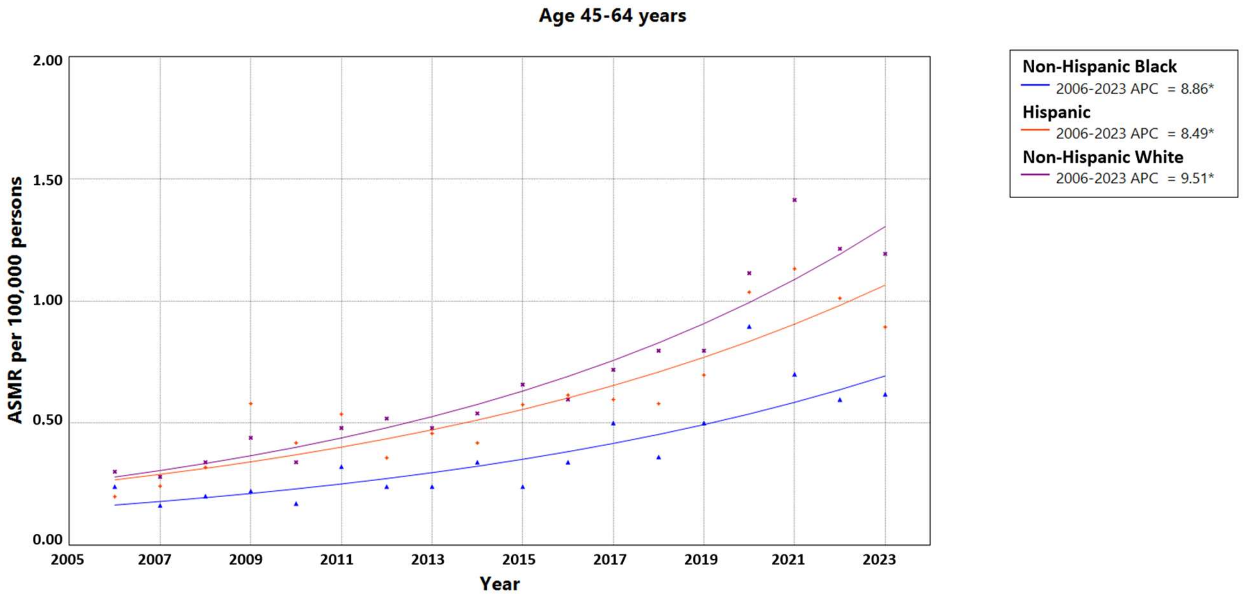

© 2025 Zhang X et al. *JAMA Network Open*.

(C)

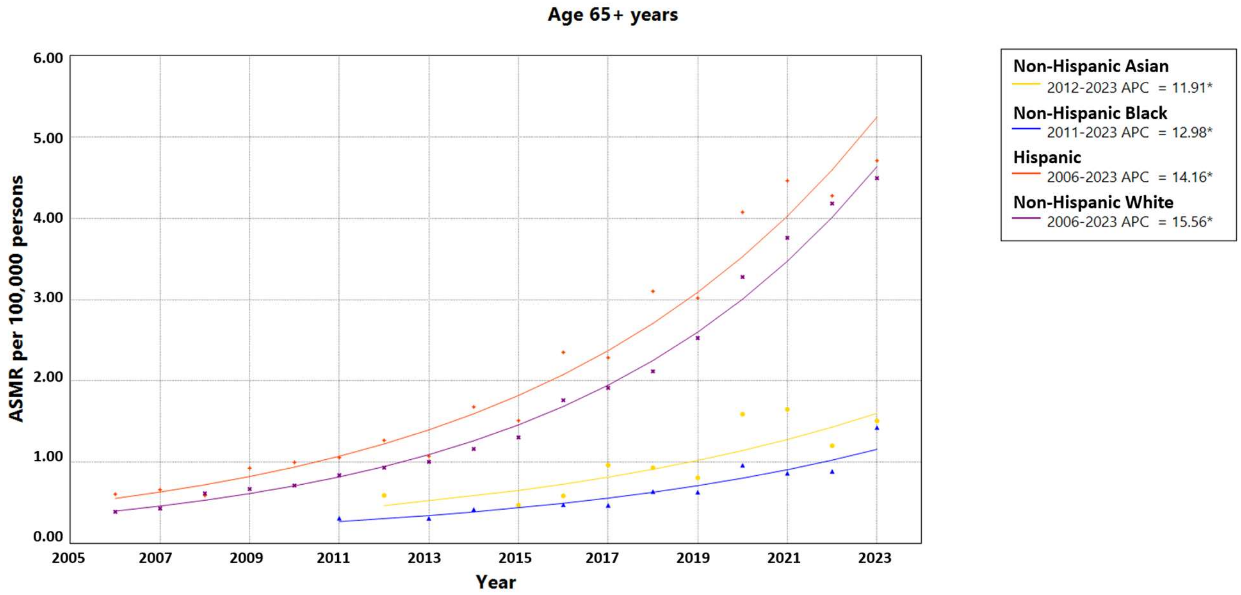

© 2025 Zhang X et al. *JAMA Network Open*.

eFigure 4. Annual Percentage Change and Age-Standardized Mortality Rate Among Decedents With MASLD as a Contributing Cause of Death, Stratified by Sex (A) Female and (B) Male Plus Race and Ethnicity (Non-Hispanic Asian, Black, Hispanic, and Non-Hispanic White) Between 2006 and 2023, Estimated by Joinpoint Regression Analysis

(A)

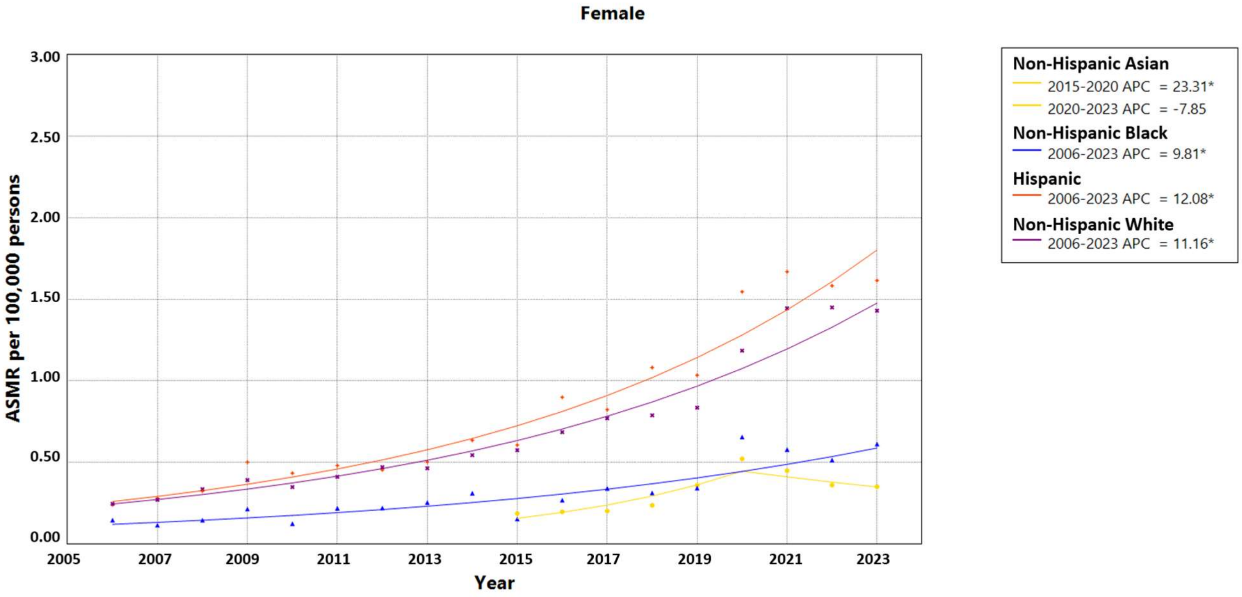

(B)

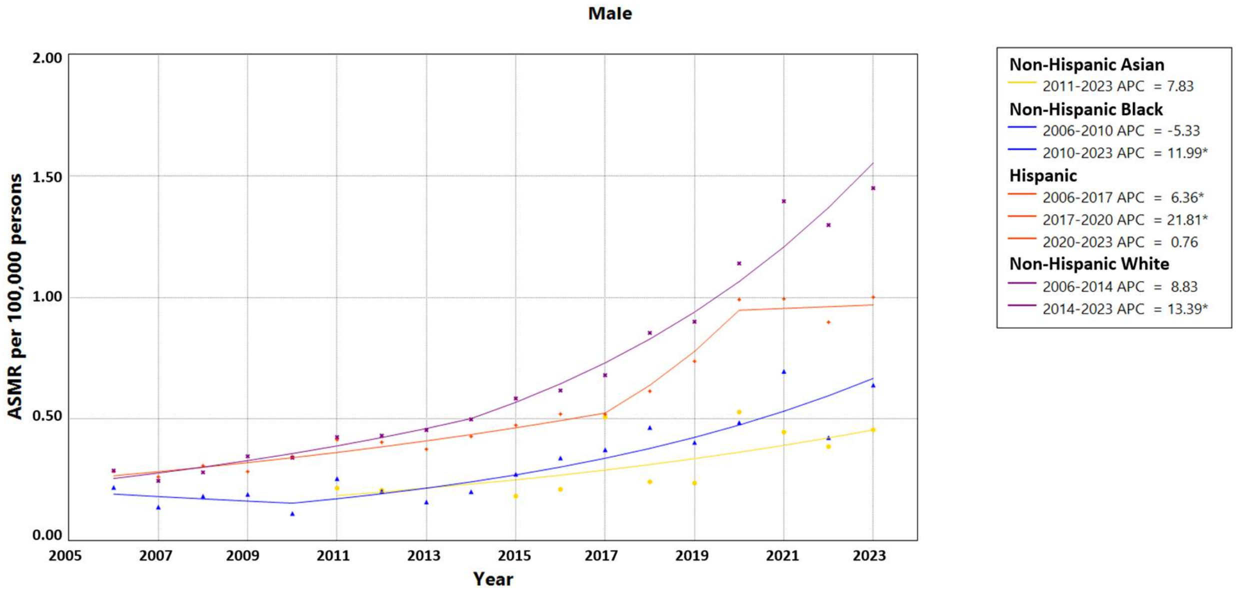

© 2025 Zhang X et al. *JAMA Network Open*.

eFigure 5. Annual Percentage Change and Age-Standardized Mortality Rate Among Decedents With MASLD as a Contributing Cause of Death, Stratified by Urbanization (A) Large and Fringe Metropolitan, (B) Medium and Small Metropolitan, and (C) Nonmetropolitan Plus Race and Ethnicity (Non-Hispanic Asian, Black, Hispanic, and Non-Hispanic White) Between 2006 and 2020, Estimated by Joinpoint Regression Analysis

Metro, metropolitan

(A)

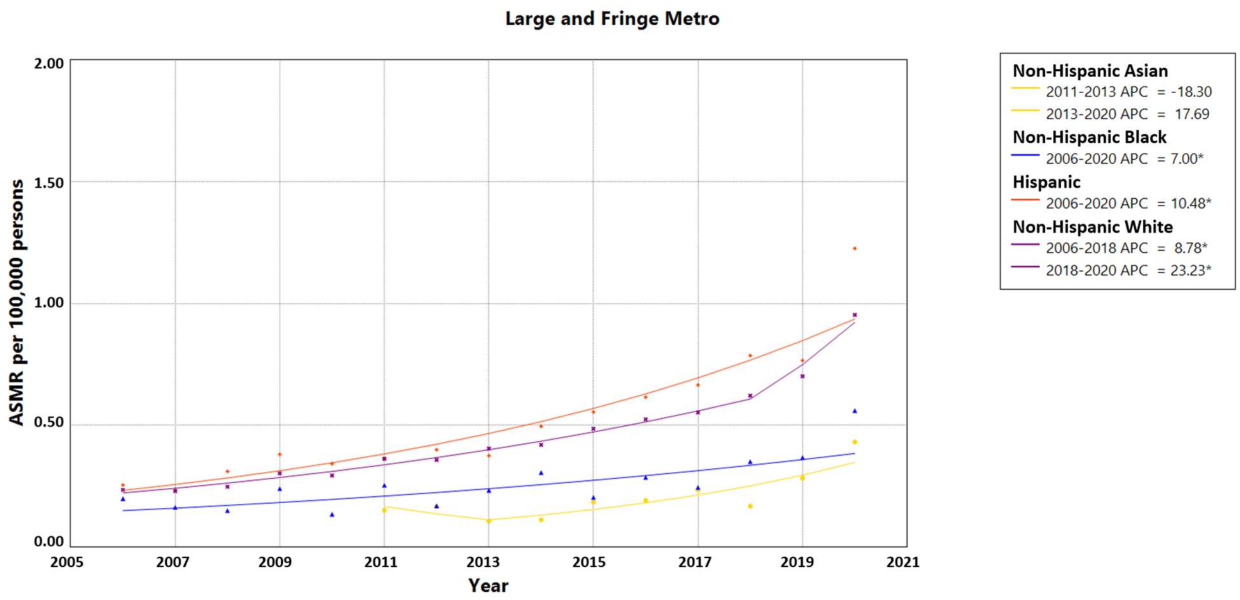

(B)

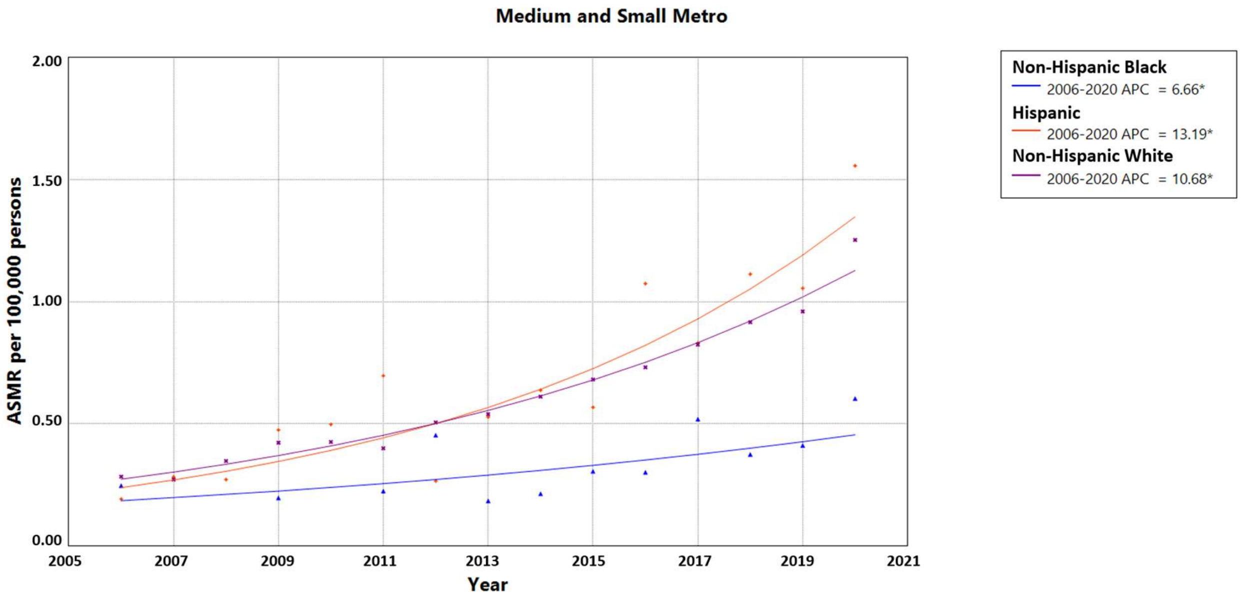

© 2025 Zhang X et al. *JAMA Network Open*.

(C)

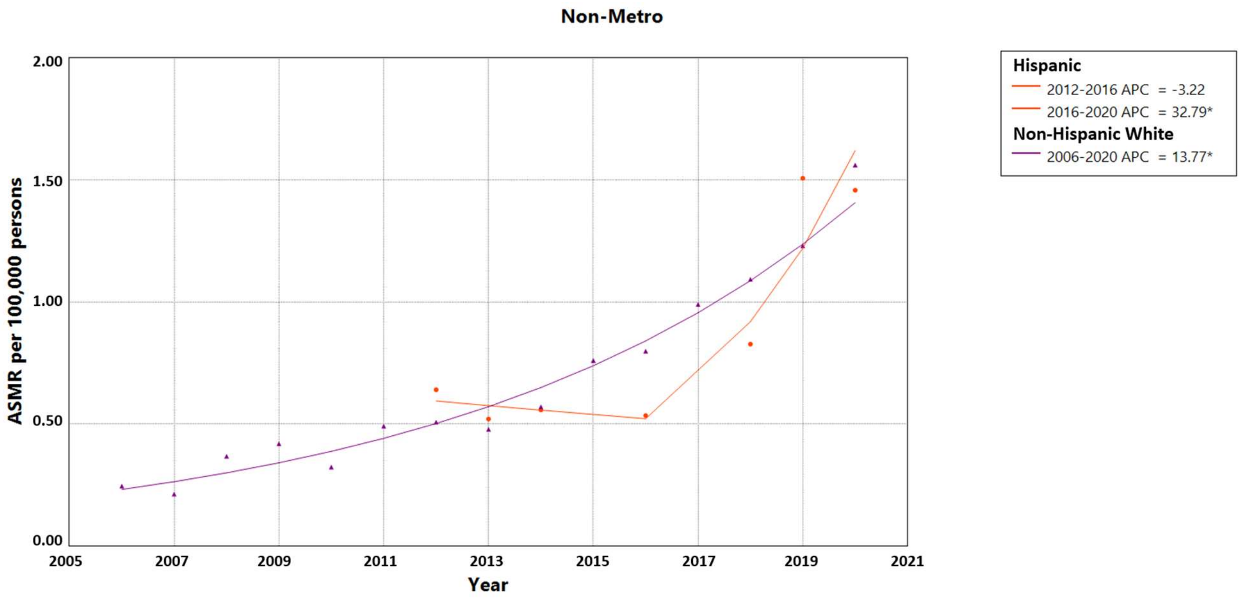

© 2025 Zhang X et al. *JAMA Network Open*.

eFigure 6. Age-Standardized Mortality Rates (ASMRs) and Projected Values for MASLD in the United States in 2006-2040, Stratified by Urbanization (A) Large and Fringe Metropolitan, (B) Medium and Small Metropolitan, and (C) Nonmetropolitan Plus Race and Ethnicity (Non-Hispanic Asian, Black, Hispanic, and Non-Hispanic White)

Metro, metropolitan

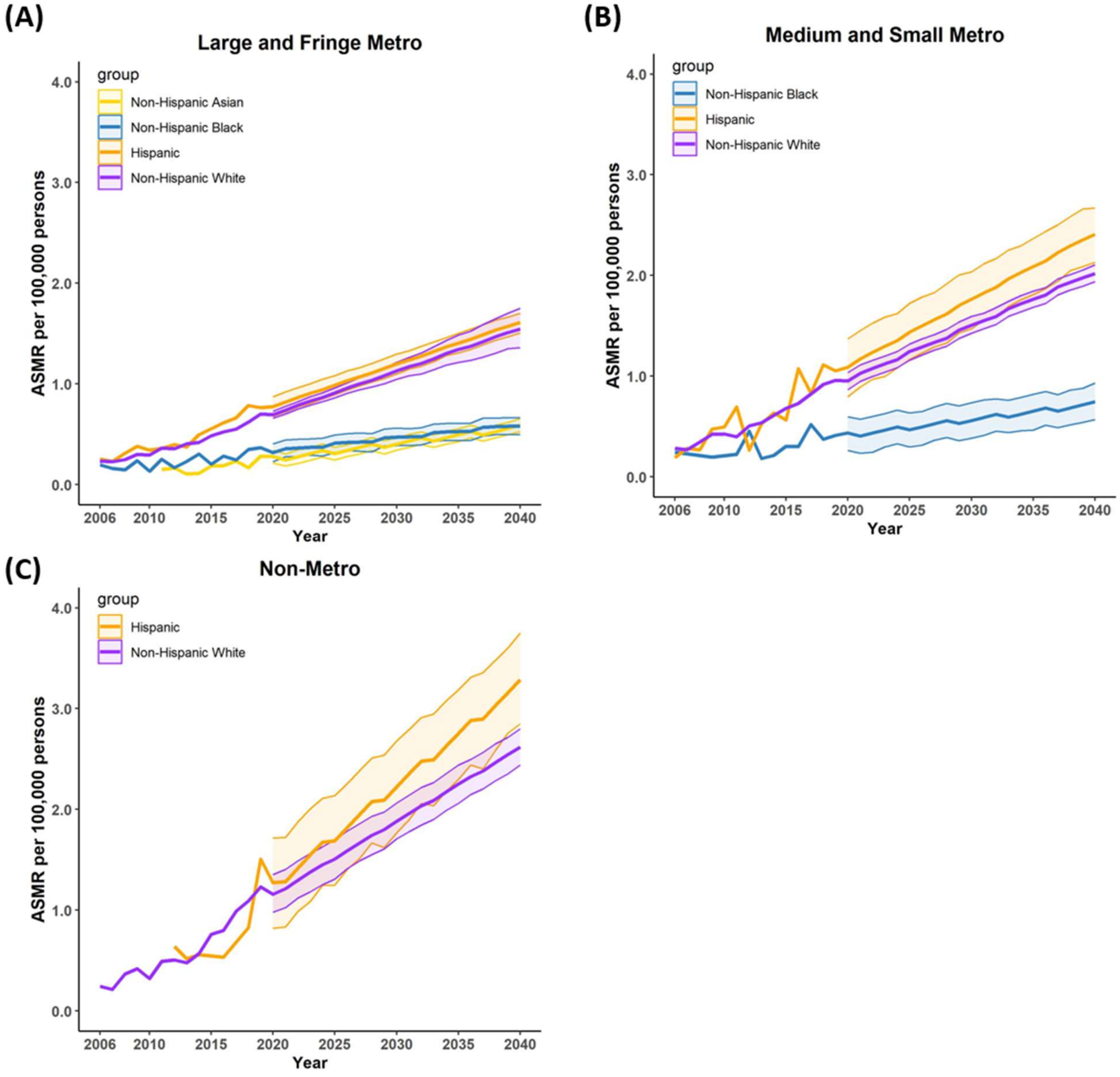

© 2025 Zhang X et al. *JAMA Network Open*.
