# Supplementary material for: Projected Trends in Metabolic Dysfunction–Associated Steatotic Liver Disease Mortality Through 2040
Source: JAMA Netw Open. 2025 Jun 17;8(6):e2516367. doi: 10.1001/jamanetworkopen.2025.16367 (PMC12175021; doi:10.1001/jamanetworkopen.2025.16367)
Supplement: Supplement 2. — Data Sharing Statement [file jamanetwopen-e2516367-s002.pdf]

Data Sharing Statement

Zhang. Projected Trends in Metabolic Dysfunction–Associated Steatotic Liver Disease Mortality Through 2040. *JAMA Netw Open*. Published June 17, 2025.
doi:10.1001/jamanetworkopen.2025.16367

Data
Data available: Yes
Data types: Deidentified participant data
How to access data: The data from this study can be accessed publicly through the National Vital Statistics System (NVSS) dataset through the Center for Disease Control and Prevention Wide-Ranging Online Data for Epidemiologic Research (CDC WONDER) website (https://wonder.cdc.gov/mcd.html).
When available: With publication

Supporting Documents
Document types: None

Additional Information
Who can access the data: All data generated or analyzed during this study will be made available only for researchers whose proposed use of the data has been approved.
Types of analyses: The data will be made available for a specified purpose and should be requested to the corresponding author and get approval.
Mechanisms of data availability: The data will be made available only when with approval of a proposal and a signed data access agreement.
